# Validation of fatty liver index as a marker for metabolic dysfunction-associated fatty liver disease

**DOI:** 10.1186/s13098-022-00811-2

**Published:** 2022-03-22

**Authors:** A Lum Han

**Affiliations:** Department of Family Medicine, Wonkwang University Hospital, Sinyong-dong 344-2, Iksan, 54538 Jeonbuk Korea

**Keywords:** Fatty liver index, Metabolic dysfunction-associated fatty liver disease, Computed tomography

## Abstract

**Aims:**

Metabolic dysfunction-associated fatty liver disease (MAFLD) is a new nomenclature for nonalcoholic fatty liver. Along with obesity, fatty liver associated with metabolic dysfunction is increasing and has become a serious socioeconomic problem. Non-invasive testing for the confirmation of MAFLD, including the fatty liver index (FLI), can be used as an alternative method for diagnosing steatosis when imaging modalities are not available. To date, few studies have examined the effectiveness and validity of FLI for diagnosing MAFLD. Therefore, this study analyzed the effectiveness and validity of FLI for diagnosing MAFLD.

**Methods:**

Medical records of men and women aged ≥ 19 years who underwent abdominal computed tomography (CT) examination at our facility between March 2012 and October 2019 were retrospectively reviewed. A comparative analysis between non-continuous variables was performed using the chi-squared test. The area under receiver operating characteristic (AUROC) curve was used to verify the effectiveness of FLI as a predictive index for MAFLD.

**Results:**

Analysis of the association between MAFLD and abdominal CT revealed that the sensitivity and specificity of FLI for diagnosing MAFLD were 0.712 and 0.713, respectively. The AUROC of FLI for predicting MAFLD was 0.776.

**Conclusions:**

Our study verified the accuracy of FLI for predicting MAFLD using CT. The FLI can be used as a simple and cost-effective tool for screening MAFLD in clinical settings.

## Introduction

Metabolic dysfunction-associated fatty liver disease (MAFLD), previously termed “nonalcoholic fatty liver disease” (NAFLD), is a new nomenclature and concept for nonalcoholic fatty liver [1]. Fatty liver associated with metabolic dysfunction is common. Along with obesity, NAFLD shows an increasing prevalence and has become a serious socioeconomic problem [2]. Inaccuracies in the terms and definitions of the heterogeneous etiology of metabolic fatty liver require reevaluation [3]. Clinically, there is a need for a concept that can more accurately reflect the management design and pathological mechanisms [2–4]. Experts are in agreement that NAFLD does not reflect metabolic dysfunction [4]. NAFLD is currently the most common cause of chronic liver disease [5] and represents one of the leading causes of cirrhosis worldwide [5]. Diagnosis of NAFLD requires an evidence of hepatic steatosis, the exclusion of excessive alcohol consumption, and the presence of other liver diseases. Owing to a lack of diagnostic criteria for NAFLD, an international group of liver specialists have recently proposed renaming NAFLD as MAFLD. The new name “MAFLD” reflects the close relationship between fatty liver and hyper-nutrition, type 2 diabetes, hypertension, dyslipidemia, and obesity. With the adoption of a positive diagnosis, MAFLD is no longer an exclusion diagnosis [6]. This makes it easier to recognize the impact of metabolic conditions on the natural history of various liver diseases, including chronic viral hepatitis and alcohol-related liver diseases [6].

Changing to MAFLD is not just a nomenclature change; classifying a liver disease as MAFLD is difficult, even if it meets the criteria for NAFLD. Even if hepatic steatosis is present, it can be classified as MAFLD only if there are elements of metabolic dysfunction meeting the criteria. The name was changed from NAFLD to MAFLD with some difficulties. Difficulties in the development of therapeutic drugs for NAFLD and biomarkers for the identification of NAFLD, specifically non-invasive testing to confirm NAFLD, are a major focus in this area. Among the most important barriers to the identification of reliable biomarkers are disease heterogeneity and the dynamic nature of histopathology, which is no longer clear with the proposed name change. As the disease phenotype becomes clearer, biomarker development is accelerated [3]. Abdominal ultrasonography is usually sufficient to detect hepatic steatosis and is the recommended primary diagnostic tool for MAFLD imaging [6]. Scores such as the fatty liver index (FLI), which can be computed with serum biomarkers, can be used as an alternative method for diagnosing steatosis when imaging modalities, such as in large-scale epidemiological studies, are not available or viable [7]. The FLI can predict NAFLD using the body mass index (BMI), waist circumference (WC), and triglyceride (TG) and gamma-glutamyl transferase (GGT) levels. The statistical significance of FLI in diagnosing NAFLD was shown in a large-scale study using ultrasonography [7]. Several European studies have reported excellent results for the FLI in predicting fatty liver [8, 9]. In studies conducted in Asia, where race and environment are different from those in Europe, studies have shown good area under the receiver operating characteristic (AUROC) curve, and FLI was statistically significant (*p* < 0.0001) in predicting fatty liver [10, 11]. Nonetheless, to date, few studies have examined the effectiveness and validity of FLI for diagnosing MAFLD. Therefore, this study analyzed the effectiveness and validity of FLI for diagnosing the newly defined MAFLD. While ultrasonography is most commonly used when diagnosing fatty liver, abdominal computed tomography (CT) has more reliable advantages, such as reproducibility, objectivity, and specificity [12]. Therefore, we used CT instead of ultrasonography to check the accuracy of FLI for diagnosing fatty liver.

## Methods

### Participants

Medical records of men and women ≥ aged 19 years who underwent abdominal CT examination at our facility between March 2012 and October 2019 were retrospectively reviewed. Patients with incomplete records, acute infectious diseases, cancer, and findings suggestive of cancer were excluded. A total of 1059 participants were included in the final analysis. Among them, 852 participants who had all records of indicators for FLI calculation were analyzed. This study followed the ethical standards outlined in in the Declaration of Helsinki; acquisition of consent from all participants was not required. We only reviewed the patients’ charts for this study and guaranteed that we would not use the information for anything other than for research purposes. Additionally, we reviewed the charts that provided only the number of participants rather than their names. This study was approved by the institutional review board of Wonkwang University Hospital (IRB approval no. 2020-06-002-002).

### MAFLD diagnosis

MAFLD was diagnosed based on one of the following criteria:

1. Overweight or obesity (defined as BMI ≥ 23 kg/m^2^) and fatty liver on a CT scan.
2. Lean/normal weight (defined as BMI < 23 kg/m^2^) and fatty liver on a CT scan with at least two metabolic risk abnormalities:

- WC ≥102/88 cm in Caucasian males and females (or ≥90/80 cm in Asian males and females)
- Blood pressure ≥130/85 mmHg or receiving specific drug treatment
- Blood pressure ≥130/85 mmHg or receiving specific drug treatment
- Plasma triglyceride level ≥150 mg/dL (≥1.70 mmol/L) or receiving specific drug treatment
- Plasma high-density lipoprotein cholesterol (HDL-C) level of <40 mg/dL (<1.0 mmol/L) for men and <50 mg/dL (<1.3 mmol/L) for women or receiving specific drug treatment
- Prediabetes (i.e., fasting glucose levels of 100–125 mg/dL [5.6–6.9 mmol/L], 2 h post-load glucose levels of 140–199 mg/dL [7.8–11.0 mmol], or hemoglobin (Hb) A1c level of 5.7–6.4% [39–47 mmol/mol])
- Homeostasis Model Assessment of Insulin Resistance scores of ≥2.5
- Plasma high-sensitivity C-reactive protein (hs-CRP) levels of >2 mg/L

3. Diabetes mellitus (according to widely accepted international criteria) and fatty liver on a CT scan.

SOMATOM Definition (Siemens Medical Solutions, Forchheim, Germany) was used for CT scans of the abdominal pelvis, and a radiologist performed the image readings. To avoid examiner bias, all data were reconfirmed by a medical imaging specialist who was blinded to the patients’ characteristics, medical history, and research objectives. Fatty liver was diagnosed when the liver attenuation value was < 40 Hounsfield units (HU) or < 10 HU compared to that of the spleen.

WC measured by the measurer and examinee using a waist tape was prone to large errors. Therefore, WC was measured according to the level recommended by the World Health Organization at the middle (half) point between the lowest rib and iliac ridge on CT images.

### Anthropometric assessment and blood test

Height and weight were measured using an automatic height scale. BMI was used to check for obesity and overweight. BMI was calculated by dividing body weight by height squared (kg/m^2^). According to the Asian criteria, BMI ≥ 23 was defined as overweight, whereas BMI ≥ 25 was defined as obesity. After keeping the blood pressure stable for at least 10 min, an automatic sphygmomanometer was used to measure the blood pressure, and the average of the two measurements was recorded.

After fasting for > 8 h, blood was collected from a vein, and the obtained blood was immediately sent to the Neodine Lab for liver function tests, complete blood cell count, blood lipid level, blood sugar level, and other serum tests. Fasting blood sugar (FBS), insulin, total cholesterol, TG, HDL-C, low-density lipoprotein cholesterol, alanine aminotransferase (ALT), aspartate aminotransferase (AST), GGT, uric acid (UA), creatinine (Cr), hs-CRP, white blood cell, hemoglobin, and vitamin D concentrations were measured.

### Clinical and lifestyle assessments

Experts at the health screening center used self-questionnaires to examine participants’ medical history and lifestyle. Participants who were diagnosed with type 2 diabetes or receiving drugs were recorded. Participants were classified as smokers and non-smokers, and those who smoked continuously for the past two years were classified as smokers. If they consumed a meaningful amount of alcohol at least once a week, alcohol consumption was marked as ‘yes’.

### FLI calculation

$$ {\text{FLI}} = \left( {e\left( {0.953 \times \ln \left( {{\text{TG}}} \right) + 0.139 \times {\text{BMI}} + 0.718 \times \ln \left( {{\text{GGT}}} \right) + 0.053 \times {\text{WC}} - 15.745} \right)} \right)/\left( {1 + e\left( {0.953 \times \ln \left( {{\text{TG}}} \right) + 0.139 \times {\text{BMI}} + 0.718 \times \ln \left( {{\text{GGT}}} \right) + 0.053 \times {\text{WC}} - 15.745} \right)} \right) \times 100$$

BMI was calculated by dividing body weight by height squared (kg/m^2^) [13].

### Statistical analyses

All statistical analyses were performed using SPSS for Windows version 26.0 (SPSS Inc., Chicago, IL, USA). A comparative analysis between non-continuous variables was performed using the chi-squared test. The AUROC curve was used to verify the effectiveness of FLI as a predictive index for MAFLD. The method developed by DeLong et al. was used to compare the FLI and AUROC. The ability of FLI to distinguish participants with MAFLD from those without MAFLD was assessed using the ROC curve. The sensitivity of the infinite determination threshold of FLI was expressed as a false positive rate, and the relevant area under the curve (AUC) was calculated. The lower limit of the AUC was considered as 0.5, and an area of > 0.5 demonstrates the effectiveness of FLI in distinguishing patients with MAFLD from those without MAFLD. The optimal cut-off point for FLI was determined using the maximum value of Youden’s J statistic [max (J = sensitivity + specificity − 1)]. The value of FLI, which corresponds to the maximum value of the Youden index, was considered the best reference point for FLI [8].

## Results

### General characteristics of patient with and those without MAFLD

The total number of participants that met the criteria was 852, comprising 574 participants without MAFLD and 278 with MAFLD. The clinical and biochemical characteristics of the MAFLD and normal groups are described in Table 1. Among the participants with MAFLD, 209 were males (75.2%), and 69 were females (24.8%); 66.2% were non-smokers; 57% had an FLI of ≥ 60, and 43% had an FLI of < 30. In the MAFLD group, BMI, systolic blood pressure, diastolic blood pressure, WC, FBS, TG, AST, ALT, GGT, insulin, and UA/Cr levels were higher than those of the control group, and HDL-C level was lower in the MAFLD group. Particularly, the FLI was significantly higher in the MAFLD group. There was no significant difference in vitamin D levels between the two groups.

**Table 1:** Characteristics of the subjects according to MAFLD

Total subjects (*n* = 852)	MAFLD		*p*-value	AUROC (95% CI)
	No (*n* = 574)	Yes (*n* = 278)		
Sex			< 0.0001	
Male	300 (52.3)	209 (75.2)		
Female	274 (47.7)	69 (24.8)		
Alcohol consumption			0.147	
N	360 (62.7)	160 (57.6)		
Y	214 (37.3)	118 (42.4)		
Smoking			< 0.0001	
N	446 (77.7)	184 (66.2)		
Y	128 (22.3)	94 (33.8)		
FLI			< 0.0001	
30 <	407 (89.8)	80 (43.0)		
≥ 60	46 (10.2)	106 (57.0)		
Age (years)	52.44 ± 10.20	52.97 ± 9.31	0.462	
BMI	23.63 ± 2.84	26.64 ± 3.12	< 0.0001	
SBP (mmHg)	121.19 ± 12.59	126.15 ± 11.77	< 0.0001	
DBP (mmHg)	74.63 ± 9.56	78.84 ± 10.02	< 0.0001	
WC (cm)	82.34 ± 8.07	89.23 ± 9.17	< 0.0001	
FBS (mg/dL)	92.3 ± 23.38	99.93 ± 26.27	< 0.0001	
TG (mg/dL)	98.8 ± 64.16	150.91 ± 143.04	< 0.0001	
HDL-C (mg/dL)	56.74 ± 12.9	52.4 ± 13.72	< 0.0001	
LDL-C (mg/dL)	119.53 ± 32.72	124.16 ± 38.03	0.082	
AST (IU/L)	30.82 ± 21.94	37.66 ± 38.42	0.001	
ALT (IU/L)	27.52 ± 24.32	40.15 ± 29.71	< 0.0001	0.701 (0.657–0.745)
GGT (IU/L)	36.76 ± 66.86	72.79 ± 215.35	0.007	0.668 (0.622–0.714)
Uric acid/creatinine	6.29 ± 1.75	6.61 ± 1.56	0.011	
hs-CRP (mg/L)	1.65 ± 3.93	2.03 ± 3.61	0.216	0.620 (0.572–0.668)
TC (mg/dL)	199.95 ± 35.45	205.11 ± 44.30	0.091	
WBC (10^3^/µL)	6019.63 ± 1735.57	6722.82 ± 1950.21	< 0.0001	
Hemoglobin (g/dL)	14.23 ± 1.50	15.34 ± 1.50	< 0.0001	0.682 (0.635–0.729)
Insulin (µIU/mL)	4.41 ± 3.02	7.76 ± 6.46	< 0.0001	0.717 (0.672–0.761)
Vitamin D (ng/mL)	18.99 ± 8.89	19.27 ± 7.86	0.688	
Fatty liver index	23.1 ± 20.75	48.63 ± 25.5	< 0.0001	0.776 (0.737–0.816)

Data are expressed as number (percentage) or mean ± standard deviation. The *p*-value was determined using the chi-squared test and independent *t*-test

*MAFLD* metabolic dysfunction-associated fatty liver disease, *SBP* systolic blood pressure, *DBP* diastolic blood pressure, *WC* waist circumference, *BMI* body mass index, *FBS* fasting blood sugar, *GGT* gamma-glutamyl transferase, *hs-CRP* high-sensitivity C-reactive protein, *TC* total cholesterol, *HDL-C* high-density lipoprotein cholesterol, *TG* triglyceride, *ALT* alanine aminotransferase, *AST* aspartate aminotransferase, *LDL-C* low-density lipoprotein cholesterol, *WBC* white blood cell

### Comparison between variables for MAFLD prediction

**Fig. 1:**
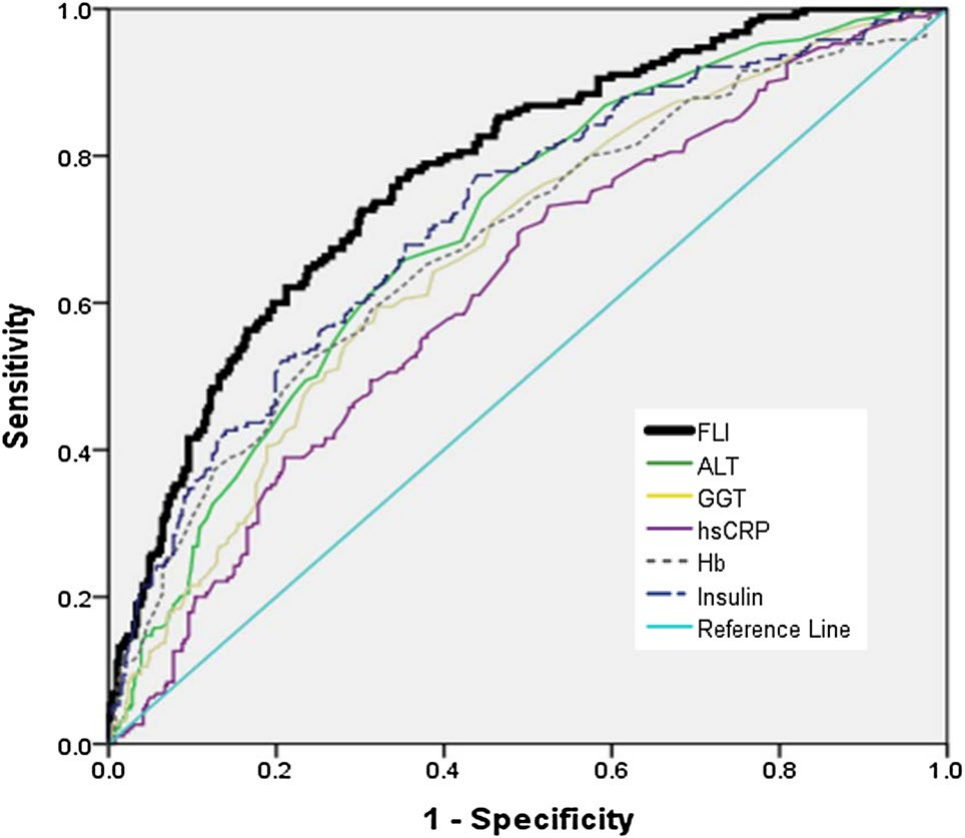
ROC curves for FLI, ALT (IU/L), GGT (IU/L), hs-CRP, Hb (g/dL), and insulin (µIU/mL). The FLI performed better than any single variable (all *p*-values < 0.0001). *FLI* fatty liver index, *ALT* alanine aminotransferase, *GGT* gamma-glutamyl transferase, *hs-CRP* high-sensitivity C-reactive protein, *Hb* hemoglobin

The AUROC of the FLI for predicting MAFLD was 0.776 (95% CI 0.737–0.816). The AUROC of the FLI was higher than that of ALT (0.694; 95% CI 0.65–0.739), GGT (0.701; 95% CI 0.657–0.745), hs-CRP (0.620; 95% CI 0.572–0.668), Hb (0.682; 95% CI 0.635–0.729), and insulin (0.717; 95% CI 0.672–0.761) (all *p* < 0.0001; Fig. 1). The cut-off value of FLI for predicting MAFLD was 30.1037, with a sensitivity of 71.2% and specificity of 71.3%.

### Concordance of MAFLD diagnosis using FLI and CT scans

We compared the correlation between the predicted MAFLD using FLI and the diagnosis of MAFLD using CT scans (Table 2). The correlation between the two was 30.1037, which is the cut-off value for the FLI obtained through the ROC curve. The results were consistent with a sensitivity of 71.2% and were statistically significant.

**Table 2:** Comparison between FLI for the prediction of MAFLD

		MAFLD No	MAFLD Yes	*p*
FLI	No	409 (71.3)	80 (28.8)	< 0.0001
	Yes	165 (28.7)	198 (71.2)	

This was analyzed with a cut-off value of 30.1037 for FLI

*MAFLD* metabolic dysfunction-associated fatty liver disease, *FLI* fatty liver index

## Discussion

In our study, we compared diagnosing MAFLD using abdominal CT to diagnosing MAFLD using FLI. We found that the sensitivity and specificity of FLI were 0.712 and 0.713, respectively, with a cut-off value of 30.1037.

MAFLD was previously known as NAFLD. The prevalence of MAFLD is increasing in Western countries owing to socioeconomic changes and the rapid transition from malnutrition to excess calorie eating habits. It is also increasing in many parts of the Asia–Pacific region, and causes public health problems. Nutritionally unbalanced and unhealthy diet and excessive energy intake relative to energy expenditure contribute to the accumulation of triglycerides in adipose tissues and in the liver. Particularly, Asians, are more likely to have visceral fat accumulation despite their low BMI [14]. South Asians in the United States have higher insulin resistance than Caucasians despite having the same or lower BMI [15]. Even non-obese and skinny Asians with NAFLD are at high risk of metabolic syndrome and type 2 diabetes mellitus [14, 16]. Larger WC and visceral adipose tissue correlated more significantly with insulin resistance and NAFLD than with a higher BMI [17]. However, although WC and visceral adipose tissue can predict MAFLD, more accurate indicators are still required and should be developed. According to the Asian Pacific Association for the Study of the Liver Clinical Practice Guidelines, abdominal ultrasonography is generally sufficient to detect hepatic steatosis and is the recommended primary diagnostic method for MAFLD imaging. Where possible, the measurement of controlled damping parameters using vibration-controlled transient elastography can be used as a more sensitive tool [6].

To evaluate NAFLD, a biopsy is performed. However, because a biopsy is invasive, a variety of imaging methods are increasingly being used. Imaging methods include both traditional and state-of-the-art technologies. Conventional imaging methods include B-mode ultrasonography, CT, and magnetic resonance imaging. The diagnostic findings of patients with NAFLD using these imaging methods are based on lipid accumulation. Conventional imaging techniques cannot assess the degree of inflammation or fibrosis. Therefore, new imaging techniques have been developed, namely, ultrasonic elastography, quantitative ultrasound-based technology, magnetic resonance elastography, and magnetic resonance-based fat quantification technology [18, 19]. In our study, fatty liver was confirmed using CT because it was only necessary to check lipid accumulation. WC was calculated using CT data to reduce the amount of errors by the examiner that occurred during WC measurement. If all imaging modalities are not available, such as in very large epidemiological studies, FLI can be used as an alternative method for diagnosing steatosis [6].

The sensitivity and specificity of FLI for diagnosing fatty liver at a cut-off value of 60 in the original study were 61% and 86%, respectively [7]. A population-based European study validated the FLI in 2652 elderly participants and showed that the FLI has a good predictive value in older individuals for both NAFLD in patients of all ages and fatty liver diagnosed by ultrasonography. The sensitivity and specificity for fatty liver were 62% and 81%, respectively. As for the prediction of NAFLD, the AUROC was 0.813 [8]. The FLI has also been used to screen individuals at risk for NAFLD. As a continuous measurement, the FLI is closely related to the presence and severity of NAFLD, as assessed using ultrasonography [8]. FLI has also been shown to be associated with cardiovascular morbidity, mortality, and incidence of diabetes [20].

The 15‐year all‐cause liver‐related, cardiovascular disease (CVD)-related, and cancer-related mortality rates were obtained to determine whether the FLI was associated with the prognosis in a population study. The FLI was found to be independently associated with liver-related mortality and was associated with all-cause mortality, CVD-related mortality, and cancer-related mortality. These associations appear to be closely related to the risks posed by the insulin resistance correlation [20]. A study involving middle-aged non-diabetic patients revealed that the intima-media thickness, increased CVD risk, and decreased insulin sensitivity were associated with high FLI values [21]. A previous study evaluated the predictive ability of two fatty liver markers (namely, the FLI and NAFLD fatty liver scores) for the onset of 9-year diabetes in the French general population. The FLI is a simple clinical tool for assessing the degree of liver fat and predicting diabetes incidence [22]. A previous study evaluated the accuracy of FLI and the optimal cut-off point for predicting NAFLD in middle-aged and elderly Chinese individuals. In this cross-sectional study, NAFLD was diagnosed using liver ultrasonography, and the accuracy and cut-off point of FLI were evaluated using each AUROC curve and maximum Youden index analysis, respectively. Thus, FLI was able to accurately identify NAFLD, and the optimal cut-off point was 30 in middle-aged and elderly Chinese individuals [11].

Jiang et al. conducted a study with 574 Chinese individuals to investigate whether FLI correlates with NAFLD and newly diagnosed coronary artery disease in a special Chinese population who underwent coronary angiography. FLI showed statistically significant results in predicting NAFLD; however, the AUROC was 0.721, which was not more effective than BMI with an AUROC of 0.728 [23]. A Korean study that verified the predictability of FLI in 376 patients. The results showed that the AUROC of BMI was the highest (0.813), followed by that of WC (0.787) and FLI (0.785) [24]. The study targeted NAFLD, and the number of participants in our study was 2.3 times that of the previous study.

Previous studies have also showed that low vitamin D levels are associated with NAFLD and are independent of factors such as metabolic syndrome [25, 26]. In an Italian study conducted on 262 participants who were referred to the Diabetes and Metabolic Disease Clinic Center, low vitamin D levels were identified to be associated with the presence of NAFLD independent of metabolic syndrome, diabetes, and insulin resistance [25]. However, in this study, the relationship between vitamin D and MAFLD was not significant. Vitamin D regulates the metabolism of free fatty acid by reducing free fatty acid-induced insulin resistance in peripheral tissues and hepatocytes. In those with vitamin D deficiency, increased free fatty acid in the bloodstream promotes fat storage in the liver and can lead to the development of NAFLD [27]. If more large-scale research is conducted, other results may be derived.

A Korean study investigated the association between MAFLD and health-related quality of life (HRQoL) to examine whether stress perception affects MAFLD [28]. The study targeted a large group using national data from the National Health and Nutrition Examination Survey. In that study, MAFLD was defined as FLI of ≥ 60 and HRQoL was assessed using EuroQol-5D (EQ-5D). The results showed that lower EQ-5D and higher stress levels were associated with MAFLD. While the study evaluated the association between mental health and MAFLD using FLI, our study verified the effectiveness of FLI in Korean subjects.

Another Korean study examined the effects of MAFLD on future mortality and cardiovascular disease (CVD) [29] using a community-based cohort study method; MAFLD was defined as FLI of ≥ 60 and at least two metabolic risk factors. The results showed that MAFLD independently increased overall mortality, and the heterogeneity of mortality and CVD risk may be determined by concomitant metabolic dysfunction such as diabetes. While the study used FLI to investigate the association between MAFLD and mortality, our study demonstrated the usefulness of FLI.

The contents of additional analysis are described below: the positive predictive value, which is the probability of an FLI of ≥ 60 in the presence of MAFLD, was 69.7%, and the negative predictive value, which is the probability of an FLI of < 30, in the absence of MAFLD, was 89.8%. Logistic regression analysis was performed to estimate the magnitude of association between the FLI cutoff point (30.1037) and the diagnosis of MAFLD through CT. The probability of MAFLD was 6.135 times higher when FLI was above the cutoff point than when it was below the cutoff point. For every 1 score increase in the FLI level, the probability of MAFLD was 1.044 times higher.

The findings of our study cannot be compared with those of previous studies due to the lack of similar studies on MAFLD; however, these results need to be discussed together with related studies in the future.

Our study has several limitations. First, it was conducted at a single center in a general hospital; hence, it cannot represent all Korean data. However, this limitation was supplemented using data from a larger number of participants, as compared to that in the previous study. Second, the diagnosis of fatty liver was not histologically confirmed through biopsy but was determined using CT.

In conclusion, our study verified the accuracy of the FLI for predicting MAFLD using CT; the AUROC of FLI for predicting MAFLD was 0.776. This suggests that the FLI can be used as a simple and cost-effective tool for screening MAFLD in clinical settings. In the future, FLI validation for predicting MAFLD should be performed in a large research group, and comparative analysis with other indices should be performed.

## Data Availability

Not applicable.
